# Supplementary material for: Patterns of Intron Gain and Loss in Fungi
Source: PLoS Biol. 2004 Nov 30;2(12):e422. doi: 10.1371/journal.pbio.0020422 (PMC532390; doi:10.1371/journal.pbio.0020422)
Supplement: Table S1 — Also available at http://genes.mit.edu/NielsenEtAl/. (4.3 MB ZIP). [file pbio.0020422.st001.zip › NielsenEtAl/html/832.html]

AN8674.1.NCU07446.1.MG02770.1.FG08593.1


```
 CLUSTAL W (1.82) Multiple Sequence Alignments - Introns Inserted


Sequence 1: MG02770.1	230 aa
Sequence 2: FG08593.1	229 aa
Sequence 3: NCU07446.1	230 aa
Sequence 4: AN8674.1	231 aa
Alignment Length: 231 aa
Number Identitical Residues: 120 aa
Alignment Score (without introns) 5616


MG02770.1 	MSQVHALSDDQ0VGQELRKMTAFIKQEAMEKAREIEIKANEEFAIEKSKLVRQETDTIDS
NCU07446.1	MSQVHALSDDQ0VGQELRKMTAFIKQEAEEKAREIQIKADEEFAIEKSKLVRQETDAIDS
FG08593.1 	MSQ-HALSDQQ0VNNELSKMTAFIKQEAMEKAREIEIKANEEFEIEKSKLVRQETDAIDS
AN8674.1  	MSQSHALSDDQ0VAGELRKMTAFIRQEALEKAREIQLKADEEFAIEKSKLVRQETAAIDT
          	*** *****:* *  ** ******:*** ******::**:*** *********** :**:

MG02770.1 	QYEKKFKQATMSQQITRSTVANKTRLKVLGARQELLDDIFDAAQKRLA-EGTKDKKKYQE
NCU07446.1	AYAKKFKQAQMSQQITRSTMANKTRLRVLGARQELLDEIFEAASAQLG-QATHDLGRYKD
FG08593.1 	QYEKKFKQATMSQQITRSTVSNKTRLKVLGSRQEMLDNIFEEAQKKLA-EGAKDKAKYQK
AN8674.1  	QYEKKFKQAAMSQQITRSTLANRTRLRVLSARQELLDDLFQEARGQISGIAAKDAKKYQE
          	 * ****** *********::*:***:**.:***:**::*: *  ::.. .::*  :*:.

MG02770.1 	ILKNLLLEGFYALHEPQMQVRARKADYDIVKSAIDAAAKEYKQKVGSEIKVTIDESNPIA
NCU07446.1	ILRDLILEGFYAMNEPELVIRARQADYDAVREAAGWASAQYKHKTDKDVKATIDAENPVP
FG08593.1 	ALKGLLLEGFFALNEPELQVRARKKDYDVVKKAIEEAAKDFKKELGKDITAKIQEDDPLP
AN8674.1  	VLKALILEGAYALNEPTVDVRARKKDKDAVKKAIGEAEKDFKVAVGKEVKINLDEEEPLP
          	 *: *:*** :*::** : :***: * * *:.*   *  ::*   ..::. .:: .:*:.

MG02770.1 	DGS2TGGVAILAGGGKIEIDNTFEARLSILNTSALPAMREALFGKNPNRKFHD
NCU07446.1	EGS2AGGIIIVGGNGKIDIDNTFEARLTLLKDSALPAMRKALFGENPNRKFFD
FG08593.1 	EGI2AGGVFIISGSGKIDIDNTFEARLKLLEESAAPAVREALFGKNPNRKFND
AN8674.1  	DDN2AGGIVITGGGGKIEINNTFEERLRLLEIDALPVVRETLFGKNANRRFHD
          	:.  :**: * .*.***:*:**** ** :*: .* *.:*::***:*.**:* *
```
